# Prognosis of Lung Adenocarcinoma Patients With NTRK3 Mutations to Immune Checkpoint Inhibitors

**DOI:** 10.3389/fphar.2020.01213

**Published:** 2020-08-12

**Authors:** Yuchun Niu, Anqi Lin, Peng Luo, Weiliang Zhu, Ting Wei, Ruixiang Tang, Linlang Guo, Jian Zhang

**Affiliations:** ^1^ Department of Pathology, Zhujiang Hospital, Southern Medical University, Guangzhou, China; ^2^ Department of Oncology, Zhujiang Hospital, Southern Medical University, Guangzhou, China; ^3^ Department of Oncology Surgery, The First Affiliated Hospital of Xi’an Jiaotong University, Xi’an, China

**Keywords:** immune checkpoint inhibitors, prognosis, lung adenocarcinoma (LUAD), NTRK3, mutations

## Abstract

**Background:**

Immune checkpoint inhibitors (ICIs) are an important treatment modality that must be considered for patients with lung adenocarcinoma (LUAD). However, ICIs are effective only in some of these patients. Therefore, identifying biomarkers that accurately predict the prognosis of patients with LUAD treated with ICIs can help maximize their therapeutic benefits. This study aimed to identify a new potential predictor to better select and optimally benefit LUAD patients.

**Methods:**

We first collected and analyzed a discovery immunotherapy cohort comprising clinical and mutation data for LUAD patients. Then, we evaluated whether the specific mutated genes can act as predictive biomarkers in this discovery immunotherapy cohort and further validated the findings in The Cancer Genome Atlas (TCGA) project LUAD cohort. Gene set enrichment analysis (GSEA) was used to explore possible alterations in DNA damage response (DDR) pathways within the gene mutation. Moreover, we analyzed whole-exome sequencing (WES) and drug sensitivity response data for LUAD cell lines in the Genomics of Drug Sensitivity in Cancer (GDSC) database.

**Results:**

Among the mutated genes screened from both the ICI treatment and TCGA-LUAD cohorts, NTRK3 mutation (mutant-type NTRK3, NTRK3-MT) was strongly associated with immunotherapy. First, significant differences in overall survival (OS) were observed between patients with NTRK3-MT and those with NTRK3-WT in the ICI treatment cohort but not in the non-ICI-treated TCGA-LUAD cohort. We then analyzed the association of NTRK3-MT with clinical characteristics and found the tumor mutation burden (TMB) to be significantly higher in both NTRK3-MT cohorts. However, significant differences in neoantigen levels and smoking history were found only for NTRK3-MT in the LUAD cohort from TCGA. Furthermore, some immune-related genes and immune cell-related genes were significantly upregulated in patients with NTRK3-MT compared to those with NTRK3-WT. In addition, NTRK3 mutation affected the deregulation of some signaling pathways and the DDR pathway.

**Conclusions:**

Our findings suggest that NTRK3-MT can predict the prognosis of patients with LUAD treated by ICIs and that it may have clinical significance for immunotherapy.

## Introduction

The revolution in immunotherapy as a new treatment landscape, specifically the development of immune checkpoint inhibitors (ICIs), has recently altered the management of nonsmall cell lung cancer (NSCLC). In particular, patients with lung adenocarcinoma (LUAD), the most common type of NSCLC, benefit most from immunotherapy. ICIs are humanized monoclonal antibodies that target programmed death 1 (PD-1), programmed death-ligand 1 (PD-L1), or cytotoxic T lymphocyte antigen 4 (CTLA-4). Numerous preclinical and clinical studies have already shown excellent survival benefits of ICIs for NSCLC patients (Brahmer et al., 2015; Fehrenbacher et al., 2016; Herbst et al., 2016; Carbone et al., 2017). However, in clinical practice, only a minority of patients respond to ICIs. Moreover, the high cost of ICIs has become one of the most severe burdens on governments and patients (Zhou et al., 2019). Therefore, the need to identify biomarkers for ICI use to improve patient selection is becoming increasingly relevant.

Fortunately, PD-L1 expression and the tumor mutation burden (TMB) have been widely studied in clinical trials, especially in NSCLC, as logical predictive biomarkers for response to ICIs (Herbst et al., 2014; Schumacher and Schreiber, 2015; Weinstock et al., 2017; Gandara et al., 2018). Both of these parameters exhibit a potential ability to predict treatment response. In addition, the mismatch repair (MMR) status, neoantigen load (NAL), and mutations in certain oncogenes (EGFR, ALK, and KRAS) (Skoulidis et al., 2015; Nathanson et al., 2017). Nonetheless, these potential predictors have several limitations, and even PD-L1 expression and the TMB have not proven to be straightforward indicative biomarkers (Brahmer et al., 2015; Rizvi et al., 2015; Cristescu et al., 2018; Garassino et al., 2018). In general, the reasons for these limitations are unclear. However, for the imperfect biomarker PD-L1, the limitations might be related to the effect of subjectivity in PD-L1 assays (Rimm et al., 2017), spatial intratumor and intertumor heterogeneity and temporal variations in PD-L1 expression, especially after chemotherapy (Ilie et al., 2016; Lim et al., 2016; Mansfield et al., 2016; Casadevall et al., 2017). Furthermore, the TMB is neither a sensitive nor specific biomarker for reliably predicting response to ICIs, despite data demonstrating a clinical benefit with respect to the objective response rate (ORR) and progression-free survival (PFS) but not overall survival (OS) (Rizvi et al., 2015; Cristescu et al., 2018). Therefore, new and reliable biomarkers to guide therapeutic strategies are urgently needed.

Several studies have demonstrated possible connections between the efficacy of immunotherapy and gene mutations. For example, clinical studies have confirmed that ICIs do not enhance OS in NSCLC patients with EGFR mutations; in other words, patients with EGFR mutations do not respond well to immunotherapy (Akbay et al., 2013; McGranahan et al., 2016; Lin et al., 2019). A study from Li et al. showed that the correlation between Tp53 mutations and tumor immunity differs among tumor types and that the Tp53 mutation status may be a negative predictor for response to ICIs in these cancers (Li et al., 2020). KRAS comutations and TET1 mutations have been demonstrated to be novel predictors for ICI response in different cancer types (Skoulidis et al., 2015; Wu et al., 2019). Overall, existing evidence shows the association between specific oncogenic mutations within tumors and sensitivity to ICIs. Therefore, the combination of driver mutations in key genes that collectively define the tumor target and markers of the environment may have better predictive value than single mutations.

In the current study, we aimed to analyze the immunotherapy-treated LUAD cohort from Samstein  et al, and we identified several novel, potentially oncogenic genes that are significantly mutated. Moreover, we evaluated the predictive value of these gene mutations in both an immunotherapy cohort and The Cancer Genome Atlas (TCGA) LUAD (TCGA-LUAD) cohort. Through this screen, we found that neurotrophin tyrosine kinase receptor 3 (NTRK3) acts as either a tumor-suppressor gene or an oncogene in the development of various cancers and that its mutation status (mutant-type NTRK3, NTRK3-MT) can predict the prognosis of patients with LUAD treated with ICIs.

## Methods

### Patient Sample Collection and Survival Analyses

To explore the importance of NTRK3 mutations in LUAD, we investigated the correlation between NTRK3 mutations and the outcome of ICI treatment in patients. First, a discovery immunotherapy cohort from Samstein  et al, which consisted of clinical and mutation data for patients receiving ICI treatment, was collected (Samstein et al., 2019). Then, after excluding five patients without mutation data (n=5), we divided the ICI treatment cohort with mutation data (n=266) into NTRK3-MT (mutant-type) and NTRK3-WT (wild-type) groups according to the nonsynonymous somatic mutation status of NTRK3 and then used Kaplan-Meier survival curves for analysis. Moreover, an R/Bioconductor package called TCGAbiolinks (Colaprico et al., 2016) was employed to download somatic mutation and OS data (n=494) from the Genomic Data Commons portal (https://portal.gdc.cancer.gov/) and the LUAD cohort from TCGA. For the latter, Kaplan-Meier survival curves were used to show the differences in OS (data from TCGA-LUAD) and disease-free survival (DFS) between patients with NTRK3-MT and NTRK3-WT. The DFS data (n=430) downloaded through cBioPortal (Cerami et al., 2012). In addition, we analyzed the association of OS with TKI-sensitive gene mutations in the ICI treatment cohort.

### Gene Mutational Signatures and Tumor Immunogenicity Analysis

All samples with somatic mutations (n=266) reported by Samstein  et al. were analyzed by targeted next-generation sequencing (NGS) and evaluated with the Memorial Sloan Kettering Cancer Center-Integrated Mutation Profiling of Actionable Cancer Targets (MSK-IMPACT) test. NAL(neoantigen load) data (n=500) from TCGA-LUAD have been reported (Gu et al., 2016). In addition, we analyzed whole-exome sequencing (WES) and drug sensitivity response data for LUAD cell lines in the Genomics of Drug Sensitivity in Cancer (GDSC) database (Yang et al., 2013). Consistent with approaches used for published data (Chalmers et al., 2017), nonsynonymous mutations in TCGA-LUAD were used as raw mutation data and divided by 38 Mb to quantify the TMB. For the ICI treatment and TCGA-LUAD cohorts, the R package ComplexHeatmap (Gu et al., 2016) was applied to visualize mutations in the genes with the top 20 mutation rates and the clinical characteristics associated with these mutations. The R package Maftools (Mayakonda et al., 2018) was used to visualize NTRK3 mutation sites.

### Comparisons of Immune Features and Drug Sensitivity Between NTRK3-MT and NTRK3-WT

A previous study showed the predictive relationship between immune cell-related genes and chemosensitivity (Newman et al., 2015). Therefore, we used CIBERSORT (Hao et al., 2018) (http://cibersort.stanford.edu/) to examine the gene expression data (RNA-seq with the Illumina HiSeq platform) for the LUAD cohort from TCGA (n=505) using TCGAbiolinks. Then, we compared the infiltration of 22 types of immune cells between NTRK3-MT and NTRK3-WT LUAD. We also examined differences between NTRK3-MT and NTRK3-WT LUAD with regard to expression of immune-related gene at the mRNA level. As reported in some studies (Thorsson et al., 2019), immune-related genes, the expression level was quantified as fragments per kilobase of exon model per million mapped fragments (FKPM) values and log2 transformed, along with their functional classification and immune-related scores, were obtained. Data for LUAD cell lines were downloaded from GDSC and compared to determine the difference in drug sensitivity between NTRK3-MT and NTRK3-WT LUAD.

### Analysis of Copy Number Alterations

Broad GDAC Firehose (http://gdac.broadinstitute.org/) was used to download Affymetrix SNP 6.0 microarray data for TCGA-LUAD (hg19; germline/potential false-positive calls were removed) and the GISTIC2.0 module of GenePattern was utilized to evaluate downloaded copy number alteration (CNA) segments (Reich et al., 2006) (https://cloud.Genepattern.org/gp/pages/index.jsf). Default parameters were used (except, for example, the confidence level was set to 0.99, and the X chromosome was excluded before analysis). The R package Maftools was used to visualize the GISTIC2.0 CNA analysis (Mayakonda et al., 2018).

### Analyses of Pathway Enrichment and the Number of Mutations in the DNA Damage Response and Repair Pathway

Differentially expressed RNAs (raw count) in the LUAD cohort from TCGA derived from TCGAbiolinks were identified using the R package edgeR (Robinson et al., 2010). The R package clusterProfiler (Yu et al., 2012) was used for gene annotation enrichment analysis. A P value of less than 0.05 was considered to indicate a significant difference in Gene Ontology (GO) terms, Kyoto Encyclopedia of Genes and Genomes (KEGG), and Reactome analyses. The data used for gene set enrichment analysis (GSEA) were obtained from Broad Institute Molecular Signatures Database (MSigDB) (Subramanian et al., 2005); gene sets of DNA damage response (DDR)–related pathways were obtained from the Broad Institute MSigDB collection (Subramanian et al., 2005). The DDR gene set was used to evaluate the number of nonsynonymous mutations in the immunotherapy (n=265) and TCGA-LUAD cohorts (n=514) and GDSC-LUAD cell lines and to identify differences in the number of nonsynonymous mutations in the DDR pathway between NTRK3-MT and NTRK3-WT LUAD.

### Statistical Analysis

Univariate Cox regression analysis was performed to identify the prognostic role of NTRK3 mutations and other common TKI-sensitive gene mutations in ICI treatment. Correlations between the NTRK3 status and the TMB, NAL, abundance of immune cells, expression of immune-related genes, age, smoking history (pack years) and number of gene mutations in the DDR pathway were assessed using the Mann-Whitney U test. Fisher’s exact test was applied to assess differences in the mutation status of the top 20 mutated genes in the immunotherapy cohort, sex and smoking history between patients with NTRK3-MT and NTRK3-WT LUAD. Fisher’s exact test was also employed to evaluate differences in sex, race, ethnicity, smoking history and clinical stage between patients with NTRK3-MT and NTRK3-WT in the LUAD cohort from TCGA. Survival curves were generated using the Kaplan-Meier method with the log-rank test. A P value of less than 0.05 was considered to indicate a significant difference. All statistical tests were two-sided. Statistical and visual analyses were carried out by using R software (version 3.6.1). The R package ggpurb was used to generate boxplots (Kassambara, 2018). CNAs with false discovery rate (FDR) of 5% as a cutoff criterion were visualized.

## Results

### Prognostic Associations of NTRK3-MT in LUAD

**Figure 1:**
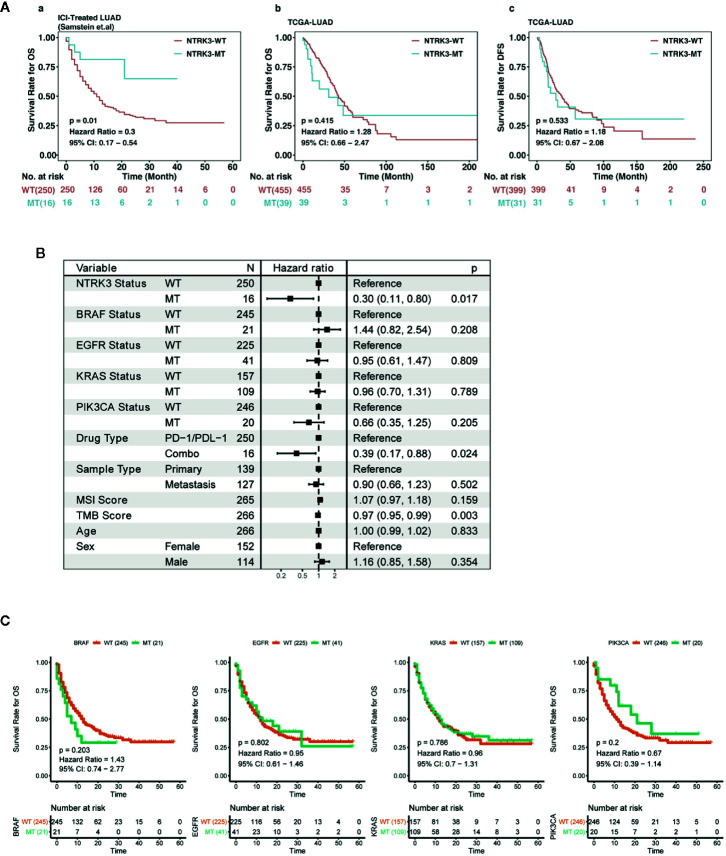
Predictive value of neurotrophin tyrosine kinase receptor 3 (NTRK3) mutation in lung adenocarcinoma (LUAD). **(A)** Kaplan-Meier analysis of overall survival (OS) for patients with NTRK3-MT or NTRK3-WT in immune checkpoint inhibitor (ICI)–treated and The Cancer Genome Atlas (TCGA)–LUAD cohorts; Kaplan-Meier estimates of disease-free survival (DFS) in the LUAD cohort from TCGA. **(B)** Forest plot displaying the association of NTRK3 mutation and other common TKI-sensitive gene mutations with ICI treatment. **(C)** Kaplan-Meier analysis of OS for patients with common TKI-sensitive gene mutations in the ICI-treated cohort.

To investigate the potential role of key gene mutations in immunotherapy, we first collected a discovery immunotherapy cohort (LUAD, n=271) from Samstein et al, comprising clinical and mutation data for patients receiving ICI treatment; mutation data of the LUAD cohort from TCGA were also downloaded. Our bioinformatic analysis workflow is shown in **Supplementary Figures S1A–C**. The Venn diagram depicted in**Figure S1D** displays the sample size of different data sets. As the schematic representation shows, screening revealed found that 16 of the 266 patients with mutation data in the above cohort had the NTRK3-MT genotype (**Supplementary Figure S1A**). Furthermore, we estimated the ability of NTRK3-MT to predict an OS benefit in both the ICI-treated and TCGA-LUAD cohorts and to predict a DFS benefit in the latter (**Figure 1A**). Univariate survival analyses indicated that the OS of the two groups, namely, NTRK3-MT and NTRK3-WT, differed significantly in the ICI treatment cohort (n =266; P=0.01; HR = 0.3; 95% CI, 0.17 to 0.54). However, Kaplan-Meier results indicated no significant difference in OS (n =494; P=0.415; HR = 1.28; 95% CI, 0.66 to 2.47) or DFS (n =430; P=0.533; HR = 1.18; 95% CI, 0.67 to 2.08) between the NTRK3-MT and NTRK3-WT groups in the non-ICI-treated TCGA-LUAD cohort. In addition, we conducted univariate Cox regression analysis to demonstrate that compared to other common TKI-sensitive gene mutations (BRAF, EGFR, KRAS, PIK3CA), only NTRK3-MT [hazard ratios (HR)=0.30, 95% confidence interval (CI)=0.11–0.80, P <0.05] correlated with a good prognosis to ICI treatment in the ICI-treated cohort (**Figure 1B**). Although we also found that the TMB score (HR=0.97, 95% CI=0.95–0.99, P <0.05) may serve as a biomarker for a good prognosis, its HR in in the ICI-treated cohort was very close to 1, which suggested that it is not a reliable predictor in this context. To date, it remains unclear whether the TMB can identify patients who may benefit from ICI treatment (Ferrara et al., 2017; Chalmers et al., 2017). In addition, survival analysis for TKI-sensitive gene mutations (BRAF, EGFR, KRAS, PIK3CA) showed no significant difference between their wild-type and mutant types in the ICI-treated cohort (**Figure 1C**). Overall, the above results clearly indicate the predominant role of NTRK3-MT in immunotherapy, which is not affected by TKI-sensitive gene mutations.

### Correlations Between NTRK3 Mutations and Clinical Characteristics

**Figure 2:**
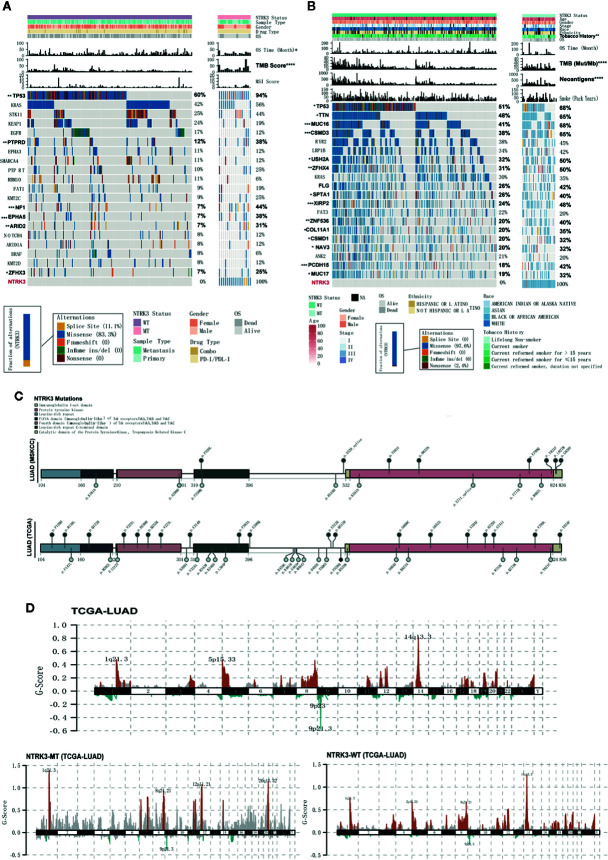
Somatic mutations and their association with clinical characteristics in lung adenocarcinoma (LUAD). **(A, B)** The clinical characteristics and top 20 significantly mutated genes are shown for the NTRK3-MT and NTRK3-WT groups in the immune checkpoint inhibitor (ICI)–treatment cohort and the LUAD cohort from The Cancer Genome Atlas (TCGA). The frequencies of each gene in each cohort are displayed on the right. **(C)** Lollipop plot of neurotrophin tyrosine kinase receptor 3 (NTRK3) mutations in both the LUAD-MSKCC panel and the LUAD cohort from TCGA. **(D)** Status of NTRK3 copy number alterations (CNAs) in the LUAD cohort from TCGA, with gains shown in red and losses in blue.

We then explored whether the NTRK3 mutation status is associated with clinical characteristics. **Figures 2A, B** show the top 20 most significantly mutated genes and the clinical characteristics in the ICI treatment and TCGA-LUAD cohorts. No significant differences were observed in the clinical characteristics of NTRK3-MT and NTRK3-WT patients treated with ICIs except for the TMB and OS. The results for the dataset TCGA-LUAD revealed that NAL, TMB and smoking history correlated significantly with the NTRK3 mutation status but that the age, sex, stage, race, and ethnicity did not. Interestingly, we also found that the mutation rate of Tp53 was higher in patients with NTRK3-MT, consistent with prior reports that Tp53 mutations are associated with enhanced antitumor immunity in LUAD (Li et al., 2020). Overall, these results highlight a potential role of NTRK3-MT as a predictive biomarker for ICI treatment. Furthermore, we annotated each mutation in NTRK3 in both the LUAD-MSKCC panel and the TCGA-LUAD in a lollipop plot (**Figure 2C**). The data from both showed that most mutations in NTRK3 occur in the protein tyrosine kinase, mainly in the immunoglobulin I-set domain and fifth domain (immunoglobulin−like) of the Trk receptors TrkA, TrkB, and tropomyosin-related kinase C (TrkC). In addition, more NTRK3 mutations in the fourth domain (immunoglobulin−like) of the Trk receptors TrkA, TrkB, and TrkC and the leucine-rich repeat C-terminal domain and catalytic domain of the protein tyrosine kinase TrkC were found in the TCGA-LUAD than in the LUAD-MSKCC panel. For the former, differences in somatic CNAs between the NTRK3 mutation statuses were assessed using GISTIC2.0. As illustrated in **Figure 2D**, amplifications on chromosomes 1, 8, 12, and 20 were enriched in the NTRK3-MT group.

### NTRK3 Mutations Enhance Antitumor Immunity and Immunogenicity

**Figure 3:**
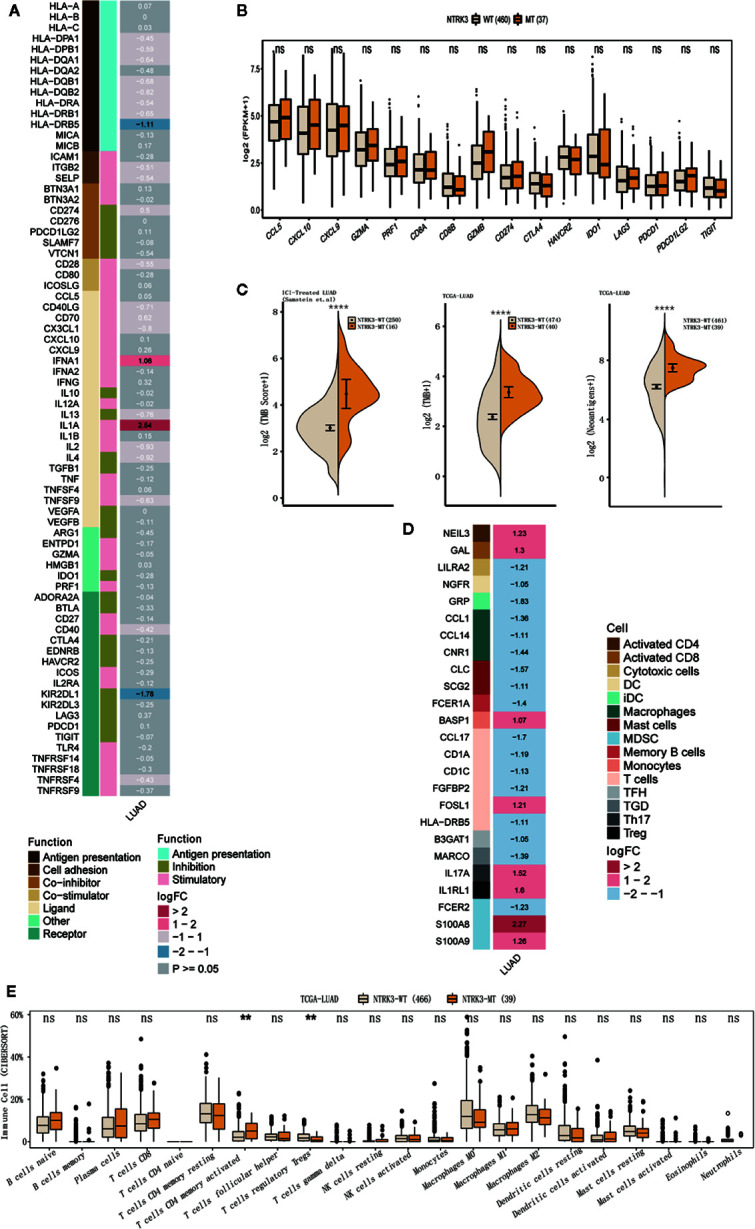
NTRK3-MT correlates with antitumor immunity and immunogenicity. **(A)** Heatmap displaying the mean differences in the expression levels of immune-related genes between the NTRK3-MT and NTRK3-WT groups in the lung adenocarcinoma (LUAD) cohort from The Cancer Genome Atlas (TCGA). From left to right, each row indicates a gene name and function, immune signature, and log2 transformed fold change (FC, fold change in the mean immune signature enrichment level or ratio). **(B)** Frequencies of stimulatory immunomodulators in the NTRK3-MT and NTRK3-WT groups of the LUAD cohort from TCGA are shown. **(C)** Comparisons of the tumor mutation burden (TMB) and neoantigen load (NAL) between the NTRK3-MT and NTRK3-WT groups in the immune checkpoint inhibitor (ICI)–treated and TCGA-LUAD cohorts. **(D)** Heatmap displaying the mean differences in the expression levels of immune cell-related genes between the NTRK3-MT and NTRK3-WT groups in the LUAD cohort from TCGA. From left to right, each row indicates a gene name, immune cell and logFC value. **(E)** Infiltration frequencies of 22 types of immune cells in the NTRK3-MT and NTRK3-WT groups of the LUAD cohort from TCGA.

We further evaluated differences in antitumor immunity and immunogenicity between the NTRK3-MT and NTRK3-WT groups in the LUAD cohort from TCGA. Some immune activation-related genes, such as interferon genes (IFNA1) and interleukin genes (IL1A), were significantly upregulated in the NTRK3-MT group, whereas the immunosuppression-related gene KIR2DL1 was downregulated (**Figure 3A**). In addition, stimulatory immunomodulators such as chemokines (CCL5, CXCL10, and CXCL9), cytolytic activity-associated genes (GZMA and PRF1) and immune checkpoint biomarkers (CTLA4, LAG3, PDCD1, and TIGIT) exhibited higher expression in the NTRK3-MT group than in the NTRK3-WT group (**Figure 3B**). Then, the TMB was evaluated in the ICI-treated LUAD and TCGA-LUAD cohorts, and as expected, it was significantly higher in the NTRK3-MT groups (**Figure 3C**), consistent with previous studies showing that tumor immunity is positively associated with the TMB. Additionally, a higher NAL correlated with NTRK3 mutation in the LUAD cohort from TCGA (**Figure 3C**).

Moreover, we investigated immune cell-related genes in the LUAD cohort from TCGA and found that genes that correlated with antitumor immunity (NEIL3, GAL, FOSL1, and BASP1) were more highly enriched in the NTRK3-MT group (**Figure 3D**). Infiltration of 22 types of immune cells was compared in the NTRK3-MT and NTRK3-WT groups of the LUAD cohort from TCGA (**Figure 3E**) and these immune cells were generally more abundant in the NTRK3-MT group.

Collectively, these results confirm that NTRK3 mutations correlate with enhanced antitumor immunity and immunogenicity in LUAD and again indicate the possible predictive value of NTRK3-MT for immunotherapy.

### NTRK3 Mutations Affect Tumor-Related Biological Pathways

**Figure 4:**
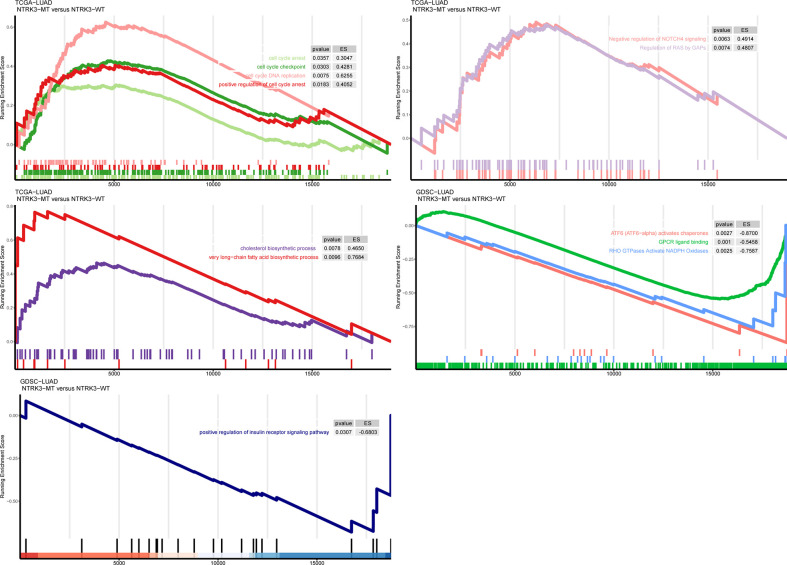
Gene set enrichment analysis **(**GSEA) of up and downregulated pathways in patients/cell lines with NTRK3-MT versus patients/cell lines with NTRK3-WT in the lung adenocarcinoma (LUAD) cohort from The Cancer Genome Atlas (TCGA) and Genomics of Drug Sensitivity in Cancer (GDSC)–LUAD cell lines.

To further explore whether NTRK3 mutations are involved in important tumor-related biological pathways and processes, we compared levels of signaling pathway enrichment between the NTRK3-MT and NTRK3-WT groups (**Figure 4**). Pathways correlating with tumorigenesis and development, such as cell cycle arrest and apoptosis, negative regulation of NOTCH4 signaling, regulation of RAS by GAPs and positive regulation of insulin receptor signaling were more highly enriched in the NTRK3-MT group. In addition, signaling pathways related to the cholesterol biosynthetic process and very long-chain fatty acid biosynthetic process pathways differed significantly in the NTRK3-MT group. However, oncogenic pathways such as ATF6 (ATF6-alpha) activate chaperones, GPCR ligand binding and RHO GTPases activate NADPH oxidases were downregulated in the NTRK3-MT group. Collectively, NTRK3-MT is associated with these pathways, reminiscent of its role in tumorigenesis.

### NTRK3 Mutations Correlate With Alterations in DDR Pathways and Drug Sensitivity

**Figure 5:**
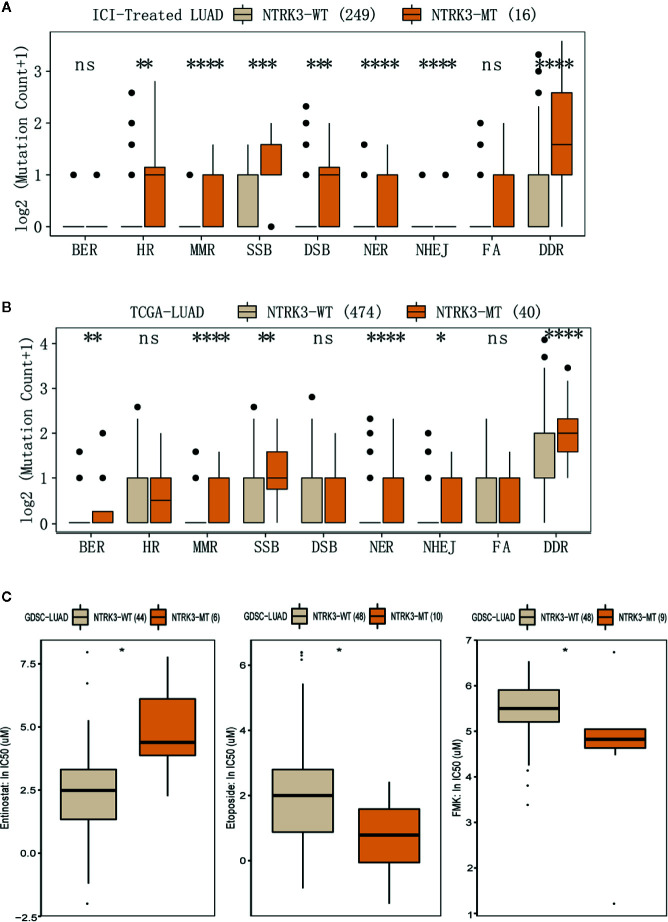
**(A, B)** Comparison of mutation counts in DDR-related pathways between the NTRK3-MT and NTRK3-WT groups in the immune checkpoint inhibitor (ICI)–treated cohort and the lung adenocarcinoma (LUAD) cohort from The Cancer Genome Atlas (TCGA). **(C)** The IC50 values of three anticancer drugs differed significantly between the NTRK3-MT and NTRK3-WT LUAD cell lines in the Genomics of Drug Sensitivity in Cancer (GDSC) database.

Reports have revealed the association of genomic instability and increased immunogenicity with the DDR pathway. To identify whether NTRK3 mutations function in DDR pathways, we evaluated alterations in various DDR pathways, such as the MMR, base excision repair (BER), nonhomologous end-joining (NHEJ), double-strand break (DSB) repair, single-strand break (SSB) repair, nucleotide excision repair (NER), and homologous recombination repair (HRR) pathways. More genomic alterations in these DDR-related pathways were observed in the NTRK3-MT group than in the NTRK3-WT group in both the ICI-treated LUAD and TCGA-LUAD cohorts (**Figures 5A, B**). We further compared the response to anticancer drugs between the NTRK3-MT and NTRK3-WT groups (**Figure 5C** and **Supplementary Figure S2**) and found statistically significant differences for the three drugs.

## Discussion

In the present study, we found that patients with NTRK3 mutations in a ICI-treated cohort showed better prognosis than patients without NTRK3 mutations. We validated this finding in a discovery immunotherapy cohort from Samstein  et al. and LUAD whole-exome data sets from TCGA. Our results indicate only patients with NTRK3-MT LUAD receiving ICI treatment had a greater OS benefit than patients with NTRK3-WT. In addition, we found that common TKI-sensitive gene mutations did not influence patient prognosis in the ICI-treated cohort. Then, we evaluated alterations in immune-related genes and immune cell-related genes in the context of NTRK3 mutation. Some genes that correlated with immune activation were found to be significantly upregulated in the NTRK3-MT group. These results demonstrate that NTRK3-MT is strongly associated with antitumor immunity and immunogenicity in LUAD.

NTRK3 is a member of the NTRK neurotrophin receptor family. NTRK3 and the other family members NTRK1 and NTRK2 encode the tropomyosin receptor kinase (TRK) family members TRKC (NTRK3), TRKB (NTRK2), and TRKA (NTRK1), respectively (Vaishnavi et al., 2015). Accumulating evidence suggests that NTRK3 is crucial to the development of not only the nervous system but also cancer. Indeed, early studies demonstrated that actionable mutations in NTRK3 occur in multiple tumors and play a vital role in tumorigenesis and treatment. For example, ETV6-NTRK3 fusions that significantly impact tumor responses have been reported in gastrointestinal stromal tumors (Shi et al., 2016). In addition, NTRK3 mutations found in human colorectal cancer promote tumor formation and progression (Luo et al., 2013). Moreover, TRK inhibitors targeting NTRK aberrations have been used in patients or clinical trials (Khotskaya et al., 2017). However, whether somatic point mutations or amplifications of the NTRK3 gene are drivers of oncogenesis has not yet been clarified. Hence, when we initially found that NTRK3 mutations in LUAD improved OS only in the ICI-treated cohort, we realized the possible critical role of this gene in immunotherapy. The subsequent findings firmly support our hypothesis.

Although ICIs are widely used in cancer treatment, predictive biomarkers for prognosis of patients to ICIs are not well established. Regardless, PD-L1 expression, TMB, NAL and microsatellite instability (MSI) status are promising biomarkers for ICIs, even though their predictive ability remains limited (Ozaki et al., 2020). Therefore, biomarkers that are more accurate and clinically useful than these biomarkers for predicting the efficacy of ICI treatment are needed. Our current study identified a new potential predictive biomarker, NTRK3-MT, in LUAD. Interestingly, a higher TMB and NAL were found in the NTRK3-MT group than in the NTRK3-WT group, a result that may confirm the nonnegligible role of NTRK3-MT in immunotherapy. Furthermore, a higher mutation rate of Tp53, which is associated with enhanced antitumor immunity in LUAD, was found in the NTRK3-MT group, possibly providing additional evidence of NTRK3-MT’s predictive capacity (Li et al., 2020).

Previous studies have indicated that NTRK3 affects multiple signaling pathways, including the MAPK and PI3K pathways, which further promote cell differentiation and affect tumor progression (Jin et al., 2010; Ozaki et al., 2020). Similarly, some effects on these pathways correlate with NTRK3 mutations. As mentioned above, mutant NTRK3 can activate the MAPK pathway in human colorectal cancer (Luo et al., 2013). Therefore, after we identified the possible predictive ability of NTRK3-MT for immunotherapy, we performed GSEA to further explore whether NTRK3-MT is involved in these signaling pathways in LUAD. As expected, pathways related to cell cycle arrest and apoptosis, negative regulation of NOTCH4 signaling and regulation of RAS by GAPs, all of which correlate negatively with tumorigenesis and development, were enriched in the NTRK3-MT group (Soriano et al., 2000; Stephen et al., 2014). Accumulating evidence has demonstrated that dysregulation of lipid metabolism contributes to the progression of various metabolic diseases, including cancers, and targeting the pathways involved in lipid metabolism has become a novel anticancer strategy. Therefore, we explored the possible pathways that regulate lipid metabolism within the context of NTRK3 mutation and found that pathways related to cholesterol biosynthetic process and very long-chain fatty acid biosynthetic process, both of which are associated with increased antitumor immunity, were significantly upregulated in the NTRK3-MT group (Mock et al., 2019; Munir et al., 2019). Similarly, the pathways ATF6 (ATF6-alpha) activates chaperones, GPCR ligand binding and RHO GTPases activate NADPH oxidases, which affect pathways promoting tumorigenesis, were downregulated in the NTRK3-MT group (Bonner and Arbiser, 2012; O’Hayre et al., 2014; Lavoie and Garrett, 2018). In addition, positive regulation of the insulin receptor signaling pathway, which increases the risk of developing multiple cancers was found to be downregulated in the NTRK3-MT group (Bray, 2002). Among these signaling pathways, we focused on alterations in DDR pathways, which have been demonstrated to be associated with an enhanced response to ICIs (Mouw et al., 2017). Consistent with this association, the major DNA repair pathways, such as the BER, SSB repair, HRR, NHEJ, DSB repair, MMR and NER pathways, were highly enriched in the NTRK3-MT group compared to the NTRK3-WT group. This finding led us to hypothesize that patients carrying NTRK3-MT may be more likely to respond to ICIs that target DDR pathways than patients with NTRK3-WT. In summary, these findings suggest that NTRK3 mutations affect multiple signaling pathways and may play roles in cancer through these pathways.

Finally, our study has several strengths. First, real-world data from an ICI-treated LUAD cohort and a non-ICI-treated LUAD cohort were used to identify the predictive ability of NTRK3-MT, which increased the credibility of the results. In addition, we evaluated differences between not only immune-related genes and immune cell-related genes but also immune-related pathways (DDR pathways) in NTRK3-MT and NTRK3-WT LUAD. Furthermore, our study compared the response to common anticancer drugs between the two groups and identified significantly different responses to three of these drugs. Nevertheless, the present study also has potential limitations. The frequency of NTRK3-MT was not high in either validation cohort; in other words, if we had used NTRK3-MT alone as a predictive biomarker, the patients with NTRK3-WT for whom ICIs were effective would have missed this critical treatment. However, combined multimarker diagnostic approaches can circumvent this limitation. In addition, preclinical studies are needed to determine whether NTRK3-MT affects the response to ICIs. Moreover, more evidence is needed to clarify the exact molecular mechanism underlying the link between ICI treatment and NTRK3 mutation.

## Conclusion

With the aim of identifying more accurate and useful biomarkers for immunotherapy, the current study identified the critical role of NTRK3-MT in LUAD patients who receive ICI treatment. Improved OS was observed in NTRK3-MT patients and those receiving ICI treatment. Antitumor immunity and immunogenicity were enhanced in the context of NTRK3 mutation. These results indicate that NTRK3-MT can effectively predict good prognosis for LUAD patients treated with ICIs. However, further clinical studies are necessary to confirm our results and to assess the value of NTRK3-MT as a predictive biomarker in immunotherapy.

## Data Availability Statement

All of the data we used in current study comes from the public databases as described in the *Methods* section.

## Author Contributions

YN, AL, and PL conceived, designed, and wrote the manuscript. WZ, TW, and RT performed the analysis and interpretation. LG and JZ initiated the study and revised the manuscript. All authors contributed to the article and approved the submitted version.

## Conflict of Interest

The authors declare that the research was conducted in the absence of any commercial or financial relationships that could be construed as a potential conflict of interest.
