# Supplementary figures and images for: Prognosis of Lung Adenocarcinoma Patients With NTRK3 Mutations to Immune Checkpoint Inhibitors
Source: Front Pharmacol. 2020 Aug 12;11:1213. doi: 10.3389/fphar.2020.01213 (PMC7434857; doi:10.3389/fphar.2020.01213)

**a**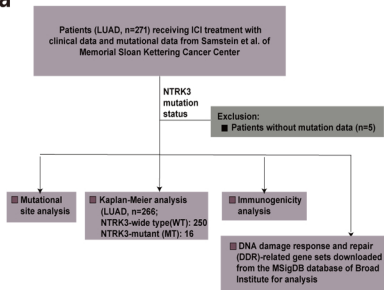**b**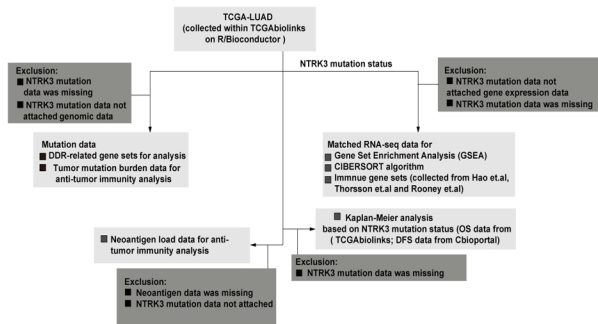**c**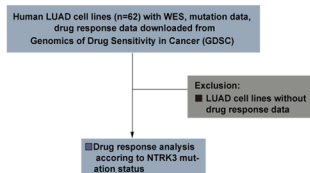**d**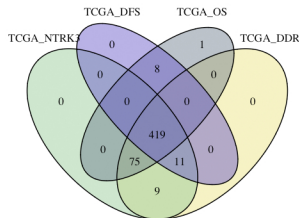

Supplement: Supplementary file 1 [file Image_1.pdf]

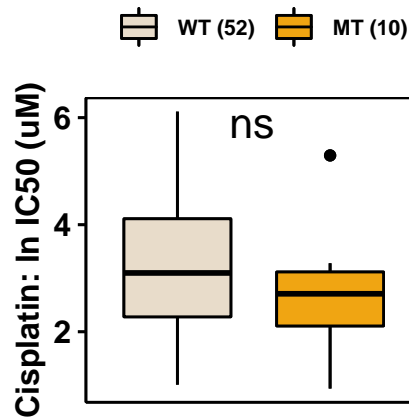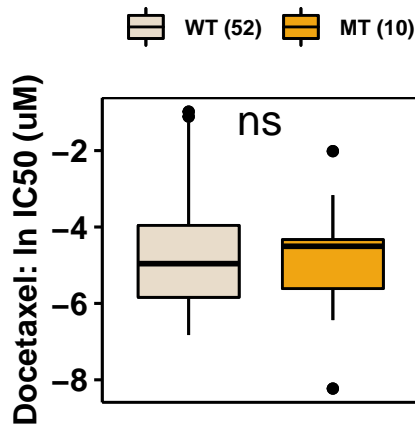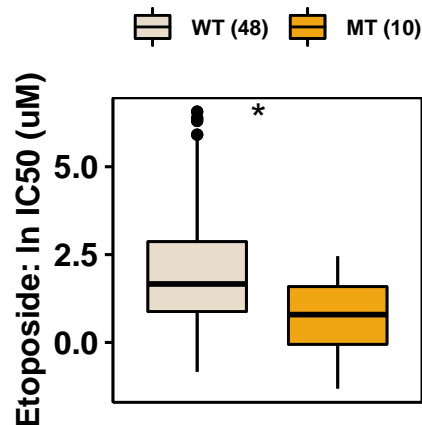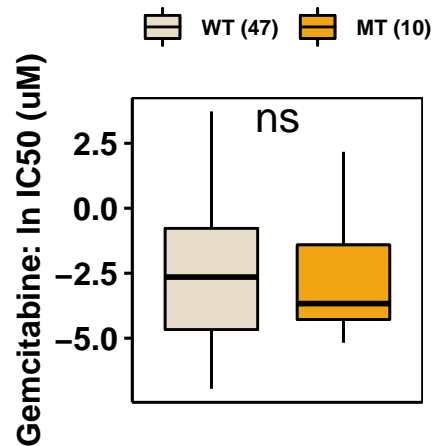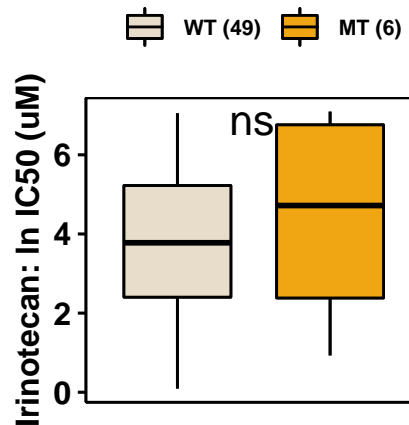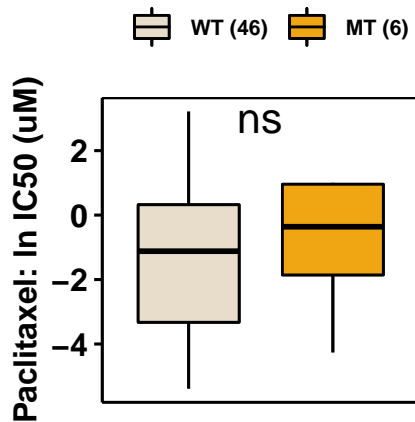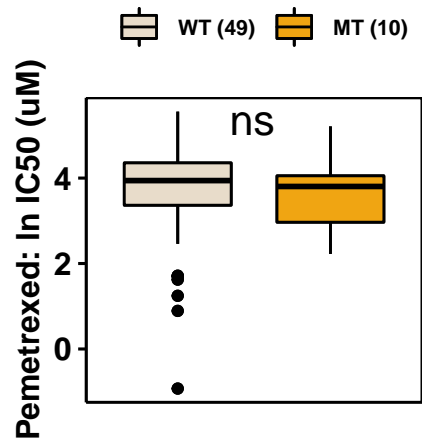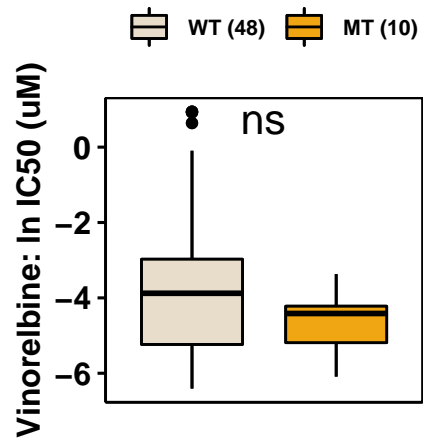

Supplement: Supplementary file 2 [file Image_2.pdf]
